# Feature-specific nutrient management of onion (*Allium cepa*) using machine learning and compositional methods

**DOI:** 10.1038/s41598-024-55647-9

**Published:** 2024-03-12

**Authors:** Leandro Hahn, Claudinei Kurtz, Betania Vahl de Paula, Anderson Luiz Feltrim, Fábio Satoshi Higashikawa, Camila Moreira, Danilo Eduardo Rozane, Gustavo Brunetto, Léon-Étienne Parent

**Affiliations:** 1 Caçador Experimental Station, Research and Rural Extension of Santa Catarina (Epagri), Epagri, Abílio Franco Street, 1500, Caçador, Santa Catarina 89501-032 Brazil; 2 Ituporanga Experimental Station, Research and Rural Extension of Santa Catarina (Epagri), Epagri, Lageado Águas Negras General Road, Ituporanga, Santa Catarina 88400-000 Brazil; 3 Department of Soil, Federal University of Santa Maria, Ave. Roraima, 1000, Building 42, Santa Maria, RS 97105-900 Brazil; 4 University Alto Vale do Rio do Peixe, Uniarp, Victor Baptista Adami Street, 800, Caçador, Santa Catarina 89500-000 Brazil; 5 State University Paulista “Julio Mesquita Filho”, Campus Registro. Registro, Av. Nelson Brihi Badur, 430, São Paulo, 11900-000 Brazil; 6 Department of Soils and Agrifood Engineering, Laval University, Quebec, QC G1V 0A6 Canada

**Keywords:** Plant breeding, Plant development, Environmental impact

## Abstract

While onion cultivars, irrigation and soil and crop management have been given much attention in Brazil to boost onion yields, nutrient management at field scale is still challenging due to large dosage uncertainty. Our objective was to develop an accurate feature-based fertilization model for onion crops. We assembled climatic, edaphic, and managerial features as well as tissue tests into a database of 1182 observations from multi-environment fertilizer trials conducted during 13 years in southern Brazil. The complexity of onion cropping systems was captured by machine learning (ML) methods. The RReliefF ranking algorithm showed that the split-N dosage and soil tests for micronutrients and S were the most relevant features to predict bulb yield. The decision-tree random forest and extreme gradient boosting models were accurate to predict bulb yield from the relevant predictors (R^2^ > 90%). As shown by the gain ratio, foliar nutrient standards for nutritionally balanced and high-yielding specimens producing > 50 Mg bulb ha^−1^ set apart by the ML classification models differed among cultivars. Cultivar × environment interactions support documenting local nutrient diagnosis. The split-N dosage was the most relevant controllable feature to run future universality tests set to assess models’ ability to generalize to growers’ fields.

## Introduction

Onion (*Allium cepa* L.) is the 4th economically most important vegetable crop grown worldwide^1^. In Brazil, onion ranks 3rd behind potato and tomato in production volume and economic value. Because onions require long days to initiate and speed up bulb swelling and reduce the maturation period^1,2^, cultivars were adapted to site conditions to reach high bulb yield and quality^3^. Rainfall also impacts onion yields^4^. High temperatures accelerate, while low temperatures delay, bulb formation^2^. The Brazilian national yield average of 26 Mg bulb ha^−1^^4^ remains far below expectations.

The law of the optimum states that production factors are used most efficiently if all combined at their optimum levels^5^, a challenging goal for growers. Alternatively, growers attempt to adjust their practices through comparisons with successful cases and relying on state recommendations from soil and tissue test results^6^. However, climatic conditions, fertilization, soil quality, irrigation, soil management and crop rotation systems are key factors of success that vary widely among agroecosystems. Uncertainty in optimum nutrient dosage often leads growers to apply ‘insurance’ fertilization against the risk of yield loss^7^. Excessive fertilization leads not only to economic loss but also to increased incidence of diseases^8–11^, product loss during storage, and environmental damage such as nitrate leaching and N_2_O emissions^12^ and surface water eutrophication by phosphates^13^. Field trials conducted under the *ceteris paribus* assumption form the backbone of sound fertilizer dosage. Such assumption no more holds at the step of assembling multi-environmental field trials due to highly variable site-specific features. Nevertheless, well-documented trials can be assembled into large databases and decrypted using powerful tools of artificial intelligence to support wise decisions on site-specific fertilization.

The traditional objective of conducting fertilizer trials is to define critical and maintenance soil test levels to “feed the plant” (sufficiency levels of available nutrients) or to “feed the soil” (basic cation sufficiency ratios; nutrient buildup and maintenance)^14–16^. In Brazil, the concept to “feed the plant” for N fertilization involved the contribution of soil organic matter content to the nitrogen budget of the agroecosystem^17^. The concept to “feed the soil” for P and K fertilization relies, respectively, on clay content and soil test P, and on cation exchange capacity (CEC) and soil test K. The clay content is assumed to be related to the soil P fixing capacity controlling P-use efficiency^18^. The CEC implied that soil test K should be maintained at a ‘High’ soil test level despite high risks of K leaching. The CEC can be computed from exchangeable cations and exchangeable acidity.

While yield-impacting features interact in agroecosystems, testing myriads of interactions between fertilizer management and environmental and managerial features would be a gigantic task. Machine learning (ML) decision trees such as random forest and extreme gradient boosting are commonly used non-parametric data-processing methods that can address multivariate interacting effects in high-dimensional databases^19–21^. On the other hand, the classical tissue test interpretation has long been criticized for not considering nutrient interactions^22^. This is especially important for onions, a high S-demanding crop, where cross-talks between sulfur and cationic micronutrients as modulated by mycorrhizae^23^ are common^24^. Nutrient interactions and cross-talks are generally represented by pairwise ratios^25,26^. The centered log ratio ($clr$) transformation is a multi-ratio that expands pairwise ratio by adjusting any nutrient level to the geometric mean across nutrients. The log-ratio transformation can control numerical biases caused by spurious correlations in the statistical analysis of compositional data^27^. The *clr* transformation thus allowed to compute means and variances unbiasedly. Nonetheless, decision-tree machine learning methods could handle nutrient interactions in onion tissue with no need for data transformation.

Fertilizer recommendations, above all nitrogen, have been puzzling for decades without agreement on which methodology is the best to balance environmental and economic outcomes^24^. We hypothesized that (1) a minimum dataset of features easy to document by stakeholders suffice to predict onion yield accurately using machine learning methods, and (2) tissue nutrient standards depend on cultivar × environment interactions. Our objective was to evaluate the capacity of machine learning models to predict onion yields and to derive tissue nutrient standards for onions under Brazilian conditions.

## Results

### Model performance to predict bulb yields

**Figure 1:**
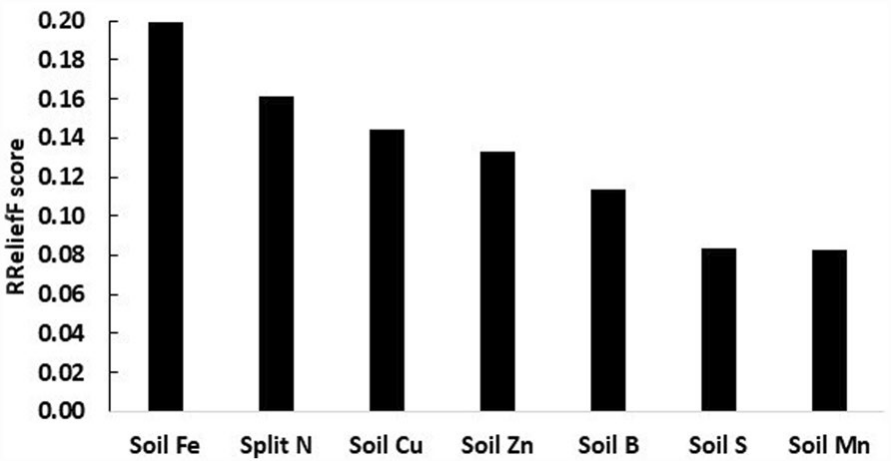
RReliefF scores indicating the most relevant features in relation to bulb yield.

Features to model bulb yields were cultivar, soil management, cropping system, previous crop, fertilization (N-P-K), soil test results (clay content, CEC, organic matter, pH, nutrients) and climatic variables (length of the growing season, rainfall, SDI, cumulated degree-days, date of crop establishment), as described in Table 6. As shown by the RReliefF scores (Fig. 1), the split-N dosage as well as soil micronutrient and S tests were the most relevant features in relation to bulb yield. Other features were weaker predictors. Soil test Fe reflects the presence of weatherable minerals (Fe) in Cambisols. Fertilizer S, B, Zn and Mn are applied at planting or sowing or as foliar sprays, hence accumulating in the soil. The Cu, Zn, and Mn applied as fungicides contribute to their accumulation in the soil.

**Figure 2:**
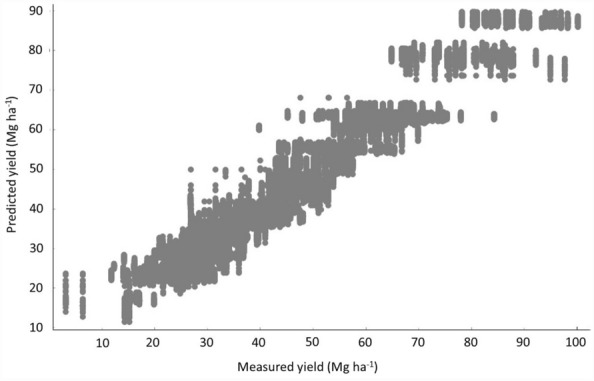
Relationship between predicted and measured bulb yields (R^2^ = 0.930).

Learners were similarly accurate (R^2^ > 0.90) to predict marketable bulb yields using either all features or a minimum data set of the most relevant features (Table 1 and Fig. 2). Non-climatic features readily available to stakeholders at the beginning of the growing season apparently sufficed to make accurate predictions of marketable yields and draw nutrient response models. The P and K dosage showed little contribution to bulb yield prediction. The N dosage was the most relevant controllable feature.

**Table 1:** Accuracy of machine learning models to predict bulb yields.

Machine learning model	Commercial yield = f(features)^a^					
	Including PK fertilizattion			Excluding PK fertilizattion		
	RMSE	MAE	R^2^	RMSE	MAE	R^2^
	kg bulb ha^−1^					
Gradient boosting	4658	3580	0.932	4716	3589	0.930
Random forest	4805	3651	0.927	4838	3653	0.926

^a^Predictors: cultivar, soil management, cropping system, previous crop, method of crop establishment, nutrient dosage (N-P-K), soil analysis (clay content, organic matter, pH, macro-nutrients, micronutrients), length of the growing season, rainfall, standardized Shannon diversity index, cumulated degree-days, date of crop establishment.

### Tissue nutrient standards

Features used to run ML classification models and compute tissue nutrient standards comprised cultivars and tissue tests. Random forest, and extreme gradient boosting returned values for area under curve (AUC) and classification accuracy (CA) at 50 Mg ha^−1^ yield cutoff (Table 2). The AUC and CA (> 90%) were high whether raw concentrations or centered log ratios were used as features, indicating that the ML models handled nutrient interactions efficiently.

**Table 2:** Area under curve (AUC) and classification accuracy (CA) for machine learning models at yield cutoff of 50 Mg ha^−1^ using raw concentrations or *clr* values as features.

Learner	Nutrient concentration values		Centered log-ratio-transformed nutrient concentration values	
	Area under curve	Accuracy	Area under curve	Accuracy
Random forest	0.994	0.940	0.996	0.959
Extreme gradient boosting	0.993	0.928	0.996	0.945

N, P, K, Ca, Mg, Fe, Mn, Zn, Cu, B, S.

**Figure 3:**
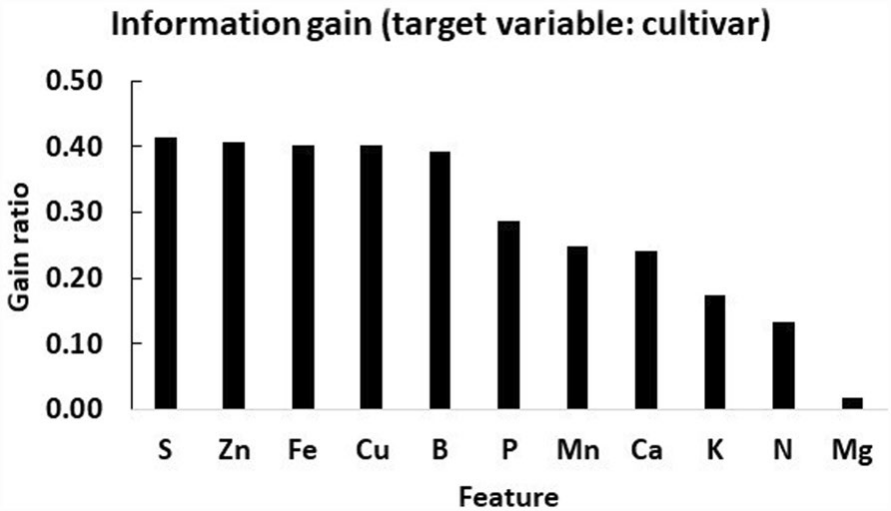
Information gain from cultivar and raw nutrient concentrations impacting onion yields.

The gain ratio showed that cultivars impacted yield more than nutrient compositions (Fig. 3). As shown by gain ratios, sulfur, phosphorus, and micronutrients impacted the ionomes of cultivars, indicating genetics × environment interactions. Indeed, several ranges of centered log ratios ($clr$) used to compute nutrient standards did not overlap among cultivars (Table 3). The back-transformed $clr$ means at high yield levels indicated differences among cultivars, especially for P, S and micronutrients. Lower and upper quartiles of nutrient concentrations among true negative specimens are presented by cultivar in Table 4. The corresponding median values of soil properties is presented in Table 5. Obviously, tissue compositions can be impacted not only by the genetic background of cultivars but also by differential soil properties.

**Table 3:** Comparison between tissue compositions of nutritionally balanced cultivars at high yield level (> 50 ton ha^−1^).

Nutrient	‘Caeté’		‘Mulata’		‘Omega’		‘SCS373 Valessul’	
	Clr	Centroid	Clr	Centroid	Clr	Centroid	Clr	Centroid
	C.I. (0.01)	g kg^−1^	C.I. (0.01)	g kg^−1^	C.I. (0.01)	g kg^−1^	C.I. (0.01)	g kg^−1^
N	(2.859, 2.935)	30.7	(3.334, 3.418)	37.2	(3.109, 3.177)	25.6	(3.172, 3.262)	26.8
P	(0.854, 1.008)	4.3	(0.995, 1.106)	3.6	(1.073, 1.137)	3.3	(1.864, 1.913)	7.1
K	(2.685, 2.749)	25.7	(3.370, 3.504)	39.5	(3.265, 3.341)	30.1	(3/234, 3.314)	28.4
Ca	(1.748, 1.813)	10.1	(2.560, 2.725)	17.9	(2.624, 2.726)	16.1	(2.011, 2.117)	8.5
Mg	(0.644, 0.712)	3.3	(0.724, 0.898)	2.9	(0.917, 1.017)	2.9	(0.864, 0.919)	2.6
S	(1.449, 1.575)	7.7	(0.823, 1.023)	3.2	(0.912, 1.013)	2.9	(1.369, 1.475)	4.4
B	(− 3.859, − 3.726)	0.038	(− 3.815, − 3.611)	0.031	(− 3.619, − 3.536)	0.031	(− 4.063, − 3.973)	0.019
Cu	(− 3.951, − 3.850)	0.034	(− 4.862, − 4.406)	0.012	(− 5.067, − 4.832)	0.008	(− 4.687, − 4.398)	0.011
Zn	(− 3.332, − 3.241)	0.063	(− 3.681, − 3.395)	0.037	(− 3.628, − 3.464)	0.032	(− 3.873, − 3.784)	0.023
Mn	(− 2.794, − 2.366)	0.129	(− 2.825, − 2.655)	0.082	(− 2.913, − 2.813)	0.063	(− 4.089, − 3.954)	0.019
Fe	(− 3.364, − 3.138)	0.066	(− 4.362, − 3.983)	0.020	(− 4.040, − 3.842)	0.021	(− 3.147, − 3.058)	0.048
Fv^†^	(6.259, 6.329)	917.9	(6.522, 6.592)	895.5	(6.701, 6.743)	918.9	(6.722, 6.789)	922.1

Values in parentheses are confidence intervals (C.I.) about centered log ratio ($$clr)$$ means at P = 0.01. Centroids are means of $$clr$$ values that have been back-transformed to familiar raw concentration values.

^†^ Filling value calculated by the difference between the geometric means and the initial filling value.

**Table 4:** Lower quartile (LQ) and higher quartile (HQ) of nutrient concentrations for nutritionally balanced onion cultivars producing more than 50 ton ha^−1^.

Nutrient	‘Caeté’		‘Mulata’		‘Omega’		‘SCS373 Valessul’	
	LQ value	HQ value	LQ value	HQ value	LQ value	HQ value	LQ value	HQ value
	g kg^−1^							
N	29.4	33.4	35.4	38.7	22.4	29.1	23.1	29.2
P	3.9	4.7	3.4	4.1	3.0	3.7	6.3	7.8
K	23.4	28.3	38.0	44.8	25.9	34.4	26.9	29.6
Ca	9.2	10.8	15.3	21.3	13.5	19.6	6.6	10.6
Mg	3.0	3.7	2.5	3.3	2.5	3.6	2.4	2.9
S	7.0	9.0	2.6	4.2	2.4	3.5	4.0	4.8
B	0.030	0.047	0.024	0.040	0.026	0.036	0.018	0.021
Cu	0.030	0.040	0.010	0.019	0.005	0.014	0.008	0.024
Zn	0.053	0.073	0.027	0.052	0.022	0.047	0.021	0.025
Mn	0.078	0.154	0.069	0.098	0.051	0.079	0.016	0.023
Fe	0.058	0.084	0.013	0.028	0.016	0.032	0.045	0.050

**Table 5:** Soil test median values at experimental sites for four cultivars at high yield level (> 50 Mg ha^−1^).

Soil property	‘Caeté’	‘Mulata’	‘Omega’	‘SCS373 Valessul’
pH	6.4	5.8	5.5	5.6
	g kg^−1^			
Clay	46	60	65	35
Organic matter	3.9	5.8	5.6	2.8
	cmol_c_ dm^−3^			
Cation exchange capacity	19.2	18.3	17.4	17.8
Ca	11.9	8.3	6.5	7.9
Mg	4.0	5.3	3.3	4.5
	mg dm^−3^			
S	22.5	15.4	20.7	39.6
K	313	207	320	117
P	18.3	3.2	7.8	17
B	1.2	0.8	1.1	1.0
Cu	4.0	13.1	7.8	0.8
Zn	10.3	2.5	2.3	2.5
Mn	3.8	18.3	27.8	8.5

## Discussion

Much efforts have been deployed by research groups in southern Brazil to reach growers’ application scale by accounting for soil test, organic matter content, clay content and cation exchange capacity^17^. In the present research, we also considered cultivar, soil and crop management, climatic indices, and tissue tests. Machine learning models using features readily available to the stakeholders were found to be accurate.

### Nitrogen recommendations

The nitrogen demand by onions was found to depend on bulb yield, cultivar, tissue nutrient levels, soil properties and fertilizer timing and placement, and thus needed to be calibrated locally^10^. Although OMC did not appear as relevant feature in relation to bulb yield as shown by its low RRelieffF score, OMC may impact N fertilizer recommendations. The N fertilization of onions in southern Brazil was adjusted to local conditions by accounting for organic matter content (OMC) (120, 100 and $\le$ 80 kg N ha^−1^ for OMC of 2.5%, 2.5–5% and > 5%, respectively) and at a rate of 4 kg N ton^−1^ for yield expectations exceeding 30 Mg ha^−1^^17^. Because OMC was included as feature in the ML model, OMC may impact the response models in future universality tests. While optimum N fertilization may vary locally from 157 to more than 200 kg N ha^−1^^28–30^, the N dosage must minimize yield loss^28^. In Cambisols of Santa Catarina, the best economic yield was reached applying 249 kg N ha^−1^ in a sandy soil of low organic matter content, and 116–142 kg N ha^−1^ in clayey soils of medium organic matter content^31^.

Boyhan et al.^32^ reported that N recommendations for onions at maximum yield in Georgia, USA, were 95–123 kg N ha^−1^ higher than the recommended N rates of 140–168 kg N ha^−1^. In contrast, maximum bulb yield of 52 Mg ha^−1^ on a Thermic Plinthic Paleudult was reached applying 263 kg N ha^−1^, as suggested by a quadratic model. However, yield differences were not significant applying 263 kg N ha^−1^ or 140 to 168 kg N ha^−1^, indicating random variation of onion yields on the plateau and high risk of overfertilization using the quadratic model. Initiating the model close to the observed optimum rate near the yield plateau can avoid that problem of overestimation. Quadratic response models initiated at zero-N depends on the flatness of the slope and may lead to over-fertilization supporting speculative ‘insurance’ decisions^33^. Controlling the trajectory of the quadratic model using an economic constraint alone, the recommended N rate for ‘Optima F1’ in Minas Gerais state, Brazil, was found to be 148 kg N ha^−1^^34^.

Although the N dosage can vary widely under different growing conditions the number of N trials was limited (25) in the present study compared the 93 and 461 multi-environmental N fertilizer trials to run ML models on potato (*Solanum tuberosum*)^35^ and maize (*Zea mays*)^36^, respectively. More trials and universality tests should be conducted to validate model outcomes in growers’ fields.

### Phosphorus and potassium recommendations

The P and K features did not appear to relevant enough to run the ML models. Irrigation and features that improves P and K diffusion in the soil increase nutrient use efficiency in tropical soils^37^. Nevertheless, the number of trials was small for P (5) and K (3) compared to N (25). As a result, more P and K trials should be conducted to support any change in state recommendations^17^. State-based recommendations integrate information from available field trials, local knowledge, and agronomic expertise.

The P dosage is generally high in tropical soils due to high soil P-fixing capacity and the limited root system of onions^38^. The clay content is representative of P fixing capacity and is integrated into the Brazilian P recommendation scheme^17^. The P_Mehlich-1_/clay ratio (Mehlich-1 extraction method) could also be used as soil test similar to the [P/(Al + Fe ratio)]_Mehlich-3_ (Mehlich-3 extraction method) currently used in North America^13,39–42^. In a low-P Humic Dystrophic Cambisol (6.9 mg P-Mehlich1 dm^−3^ and 24% clay), onion responded linearly to P fertilization in the range of 0 to 210 kg P ha^−1^ at yield levels up to 45 ton ha^−1^^28,38^. In a medium-P dystrophic red-yellow Latosol (9.1 mg P-Mehlich1 dm^−3^ and 26% clay), onion responded non-linearly to added P up to ≈131 kg P ha^−1^ at yield levels of 36–40 ton ha^−1^^43^. In a low-P dystrophic red-yellow Latosol (23.8 mg P-Mehlich1 dm^−3^), onion responded non-linearly to added P in the range of 27 to 80 kg P ha^−1^ at yield levels of 75–76 Mg ha^−1^^44^. Those results may fit state recommendations^21^ if the yield level is considered. The split of P fertilization may improve P-use efficiency, especially in high P-fixing soils^45^. On the other hand, onion P uptake is facilitated by the positive effect of irrigation on the P diffusion process^46^. The P dosage using the efficiency coefficient of fertilizer P alone^18^ and disregarding water supply that facilitates P diffusion in the soil could thus lead to overfertilization^37^. Moreover, colonization of onion roots by arbuscular mycorrhiza fungi (AMF) can regulate the P uptake by exploring a larger volume of soil^47^.

The K dosage is most often prescribed to ‘feed the soil’ depending on the selected maintenance soil test K level and the CEC. In a soil containing 77 mg K-Mehlich1 dm^−3^ and showing CEC of 7 cmol_c_ dm^−3^, onion crops responded non-linearly to added K up to 75 kg K ha^−1^ at yield level of 66 Mg ha^−1^^43^. In a high-K Red-Yellow Argisol showing 97–109 mg K-Mehlich1 dm^−3^ and CEC of 7 cmol_c_ dm^−3^, onion responded non-linearly to added K up to 150 kg K ha^−1^ to reach yield levels of 46–54 Mg ha^−1^^48^. Those results may fit state recommendations^21^ if the yield level is considered. In a work carried out in Santa Catarina state with cultivar Empasc 352 Bola Precoce, 86.5 kg K ha^−1^ was taken up by the onion crop at yield level of 37 Mg ha^−1^, accumulating 2.3 kg of K per Mg^47^. While soil K supply capacity also depends on soil mineralogy^49^, the K release from minerals that contributes to plant K uptake requires conducting fertilizer trials^50^. Large discrepancies may thus occur among K recommendation systems.

### Tissue diagnosis

In the present study, we suggested ranges of tissue nutrient levels as nutrient standards to conduct nutrient-by-nutrient diagnosis. S-É Parent^51^ suggested using a concept of reachable hyper-islands or ‘*hyper-blobs*’ each representing multivariate combinations of successful conditions compared to those of defective specimens. Using KNN as machine learning model, compositional proximity was shown as an Euclidean distance between the composition of the diagnosed specimen and that of its successful neighbors^52^. Benchmark *blobs* were also called ‘Enchanting Islands’^53^, ‘Humboldtian loci’^54^, and ‘Ilhas Encantadas’ in Portuguese^55^. This emphasizes the need to diagnose tissue nutrient compositions holistically rather than separately^56,57^.

### Need for large and diversified databases

Large and diversified experimental and observational data sets must be acquired by stakeholders to cross-over the numerous combinations of crop-impacting features in onion agroecosystems^57–60^. Kyveryga et al.^33^ stated that the development of new nutrient calibration procedures has been limited by the inability in the past to collect a sufficient number of yield responses to enable calculating reliable economic optimum rates. To follow-up on model predictions, universality tests are needed to verify the reliability of model outcomes in growers’ fields^36,61^. The prediction of N dosage can be conducted as shown in S4 by providing the site-specific feature and drawing a response curve predicted from those features. Such tests require close collaboration with growers to facilitate the acceptance of a site-specific fertilizer program and update the database.

Precision farming technologies could allow collecting trustful data at low cost in growers’ fields. Efforts to develop technological tools of precision agriculture for site-specific fertilization have been limited by non-specific state-based fertilizer recommendations. For some high-valued crops like maize, the nitrogen dosage can be adjusted to local factors using ML methods^36^. Observational and experimental data sets could be further combined and processed by machine learning to customize nutrient management for a given set of controllable and uncontrollable features^62^. In this paper, accurate ML learners processed a minimum data set to support wise decisions for the feature-specific N fertilization in onion agroecosystems of southern Brazil.

## Conclusions

This paper addressed onion nutrient management at local scale. We assembled the results of fertilizer experiments conducted between 2007 and 2020 in Santa Catarina state, the major onion production region in Brazil. We showed that decision-tree machine learning models can return accurate yield predictions under a set of easy-to-collect features. Key features available to growers before planting or seeding included cultivar, soil management, cropping system, previous crop, fertilization (N-P-K), soil test results (clay content, CEC, organic matter, pH, nutrients) and date of crop establishment. The RReliefF scores revealed that split-N dosage as well as soil test S and micronutrients were the most relevant features to predict onion yield. The accuracy of the regression models reached R^2^ > 90% using random forest and extreme gradient boosting. The N dosage was the most relevant controllable feature to run universality tests in growers’ fields to assess the ability of ML model to generalize.

The accuracy of the classification models also reached R^2^ > 90% using random forest and extreme gradient boosting. The cultivar and tissue nutrients impacted bulb yield, allowing to develop cultivar-specific nutrient standards. Sulfur and micronutrients were the most relevant features to differentiate onion cultivars, indicating cultivar × environment interactions. It is thus advisable to conduct tissue diagnosis considering agroecosystem-specific nutrient standards to reflect cultivar × environment interactions. To set apart genetics and environment, feature-specific cultivar ionomes should be determined in comparable agroecosystems. However, such agroecosystem nutrient standards would require larger and more diversified databases than the one used in this study.

## Material and methods

### Experimental setup

**Figure 4:**
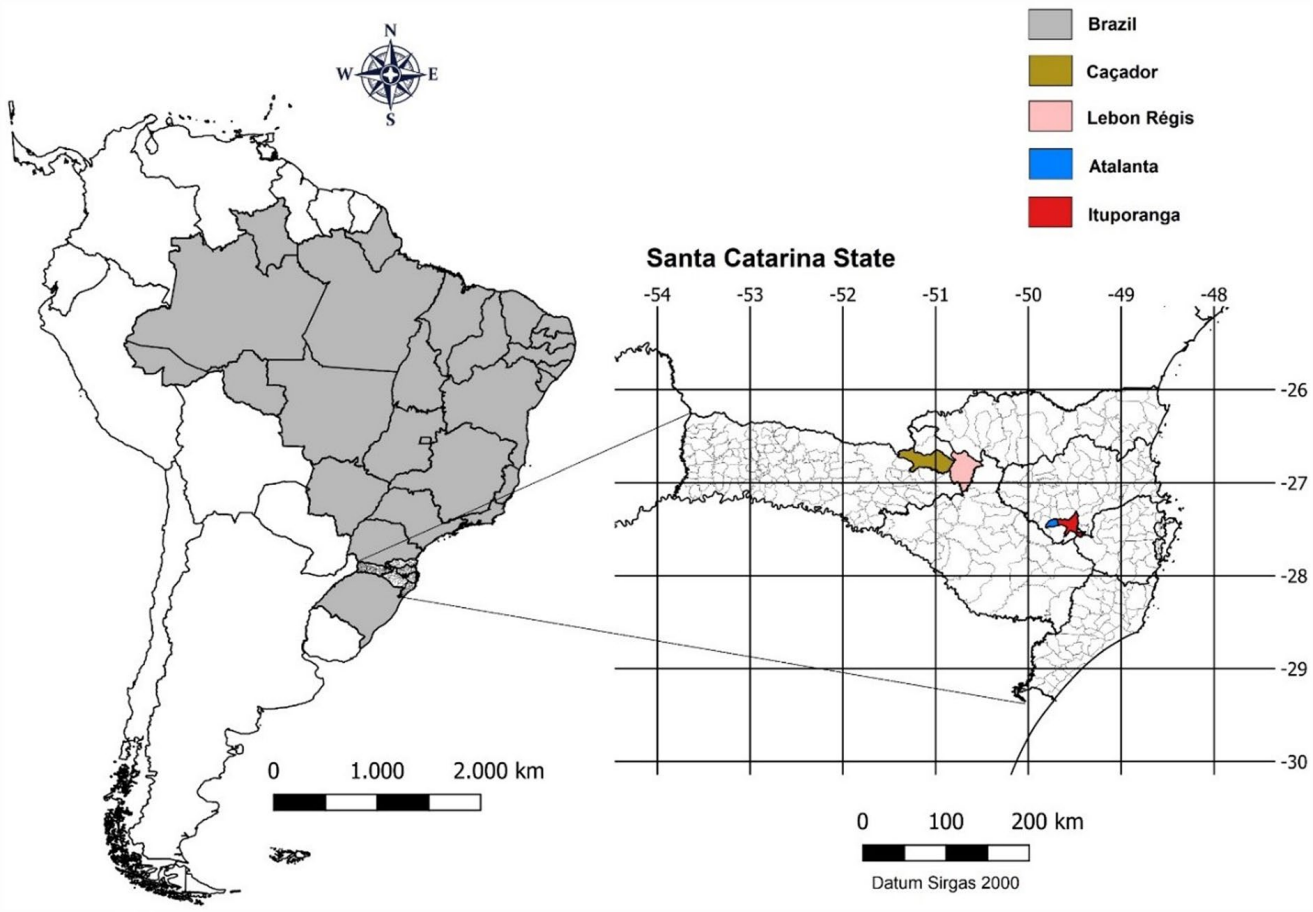
Geographic location of the Caçador, Lebon Régis, Atalanta and Ituporanga municipalities in Santa Catarina State, southern Brazil, where onion fertilizer trials were conducted. Map created by QGis software^65^ version 3.34.3.

Fertilizer trials were conducted from 2007 to 2020 in the municipalities of Ituporanga, Atalanta, Lebon Régis and Caçador, Santa Catarina state, Brazil (Fig. 4). The soils of the region are Cambisols, also classified as Nitossolo Bruno Distrophic^63^, and Typic Hapludox^64^. The subtropical climate is mesothermic and humid with mild summers.According to Köppen’s classification, the climate is classified as Cfa in Ituporanga and Atalanta, and as Cfb in Lebon Régis and Caçador.

### Climatic data

Daily precipitations as well as minimum and maximum daily temperatures were obtained from the EPAGRI^66^ meteorological station closest to the trial. Temperature indices were the minimum and maximum seasonal temperatures and the cumulated degree-days with base temperature of 5 °C for cold crops^64^. Rainfall distribution was estimated by the standardized Shannon diversity index (SDI) as follows^65^:

$$SDI=\frac{-{\sum }_{i=1}^{n}{p}_{i}\times ln\left({p}_{i}\right)}{ln\left(n\right)}$$

where ${p}_{i}$ is the fraction of daily rainfall (RAIN) to the rainfall cumulated during the growing period (PPT), i.e. the daily RAIN/PPT ratio, and $n$ is the length of the growing season; SDI = 1 implied that rainfall was uniformly distributed during the indicated period (equal daily amount of rainfall over the selected period); SDI = 0 implied that rainfall was unevenly distributed (total rainfall concentrated in 1 d). Where ${p}_{i}=0$, ${p}_{i}\times ln\left({p}_{i}\right)=0$. Crops were sprinkler irrigated.

### Experimental setup

There were 26 N trials, five K trials and three P trials, totaling 1182 observations (Supplementary Material S4). Treatments were arranged as randomized block designs with four replications. In Ituporanga and Atalanta, plots were 4 m long and 3 m wide, and comprised eight rows spaced 35 cm apart. Transplants were spaced 8 cm apart on the row. The population of transplants was approximately 375,000 plants ha^−1^. Bulbs were harvested in five internal rows 4-m long. In Caçador and Lebon Régis, plots were 5 m long and 2.7 m wide, and comprised nine rows spaced 30 cm apart. Plants were spaced 5.5 cm on the row. The population of seeded onions was 600 000 plants ha^−1^. Bulbs were harvested at leaf sagging in three double line, 5-m long rows, per plot. The bulbs were left on the field for a pre-curing period of one week, then bagged and stored for weighing and sizing. Bulbs were classified as commercial, non-commercial and harvest loss. Marketable bulbs included #2 (< 50 mm), #3 (50–70 mm), #4 (70–90 mm), and #5 (> 90 mm) bulb categories^67^. Bulbs showing secondary growth or damage were classified as non-marketable.

### Fertilizer treatments

The N, P and K treatments were applied separately at increasing rates at each experimental site. The N rates varied from 0 to 370 kg N ha^−1^ split-applied 45, 80, 110, and 130 days after seeding, 20, 30, 30 and 20% of N broadcast-applied, respectively, or 35, 60, and 85 days after transplanting, 30, 40 and 30% of N broadcast-applied, respectively. The P rates ranged from 0 to 349 kg P ha^−1^. The K rates varied between 0 and 667 kg K ha^−1^, split-applied together with the N. Where the rates of N, P and K were varied, the rates of the other nutrients were fixed following state recommendations^17^. Fertilizers were in granular form.

The sources of N were ammonium nitrate, urea, ammonium sulfate, algae-coated ammonium sulfate (29% N, 5% Ca, 2% Mg, 9% S, and 0.3% B), azoslow (organo-mineral fertilizer containing 20% C and 29% N as urea and hydrolyzed proteins) or poultry manure (pH of 7.8, 15.9% moisture, 3.5% N, 3.1% P, 2.7% K, 37 mg Cu kg^−1^, 43 mg Zn kg^−1^, 73 mg Mn kg^−1^, and 1160 mg Fe kg^−1^). The source of N fertilizer may differ among trials. However, we assumed that differences among mineral N sources were negligible due to the rapid conversion of ammonium to nitrate in agricultural soils^68^. The P and K treatments were applied as triple superphosphate and potassium chloride^17^. The N and K were split at up to four occasions during the season, i.e., at planting and 35, 60, 85 or 90 d later for transplants, or at planting and 45, 80, 110 or 130 d later for seeded onions^17^. The P was applied entirely at planting.

### Soil analysis

Soils were sampled in the 0–20 cm layer 45–60 days before planting across the experimental area, then composited. Soils were dried in a forced-air oven at 65 °C then ground to less than 2 mm. Chemical analyses were conducted as follows^17^: pH in 1:2.5 soil-to-water volumetric ratio, clay by sedimentation, Mehlich-1 extraction for P and K, and EDTA-extraction for cationic micronutrients. Elements were quantified by colorimetry for P and B, flame photometry for K, turbidimetry for S, and atomic absorption spectrophotometry for Ca, Mg, Cu, Fe, Mn, and Zn. Total carbon was quantified by dichromate oxidation (Walkley–Black procedure) then multiplied by 1.724 to derive organic matter content. Base saturation was computed as the sum of cationic species (cmol_c_ kg^−1^) divided by CEC computed as the sum of exchangeable cations and acidity. Exchangeable acidity was assessed as follows^69^:

$$\left(Exchangeable\; acidity\right)=10exp\left(7.76+1.053{\times pH}_{SMP}\right),\;\; {\text{R}}^2 = 0.98$$

### Tissue analysis

After planting, leaf analysis, based on appropriate sampling methods and correct interpretation of analytical data, is a reliable tool for assessing the nutritional status of perennial plants and their response to fertilizers^69^. Ten young fully expanded leaves were collected in each plot at the beginning of plant differentiation into bulb^17^, i.e. 70 to 75 d after transplanting and 115 to 128 d after sowing, depending on year and cultivar. Tissue samples were composited per plot for chemical analysis. The leaves were cleaned gently under distilled water then dried at 65 ± 5 °C and ground to less than 1 mm. Total N was quantified by micro-Kjeldahl. Tissue samples were digested in a mixture of nitric and perchloric acids then analyzed by colorimetry for P and B, flame photometry for K, turbidimetry for S, and atomic absorption spectrophotometry for Ca, Mg, Cu, Fe, Mn, and Zn^70,71^.

### Statistical analysis

#### Log ratio transformation

Concentrations are parts of a compositional vector constrained to the compositional space^68^ such as 1000 g kg^−1^ for tissue tests. The compositional space for cationic species could also be defined as cmol_c_ kg^−1^ and constrained to CEC. Conducting parametric statistical analyses using raw concentrations produces numerical biases that may lead to sums of components in statistical results that differ from measurement unit (e.g., sums of sand + silt + clay different than 100% after conducting ANOVA). Moreover, ignoring nutrient interactions may decrease the accuracy of nutrient diagnosis using parametric methods^37,55^.

In contrast, $clr$ values are relative expressions allowing compositions to move from the constrained compositional space to the unconstrained real space ($\pm \infty$) that is required to run statistical analyses. Nutrient concentrations are constrained to the measurement unit using a filling value ${F}_{v}$ computed by difference as follows using a measurement unit in g kg^−1^:

$${F}_{v}=1000-\sum \limits_{i=1}^{D}{c}_{i}$$

where *D* is the number of parts including the filling value, and ${c}_{i}$ is the concentration of each nutrient and the filling value. The centered log ratio centers any concentration against the geometric mean across parts [$clr=ln\left({x}_{i}/G\right)$], hence accounting for all pairwise ratios that reflect nutrient interactions and cross-talks^24,69^, as follows for nitrogen (N):

$$\begin{aligned} {clr}_{N}&=ln\left(\frac{N}{G}\right)=ln\left(\frac{N}{{\left(N\times P\times K\times Ca\times Mg\times S\times B\times Cu\times Zn\times Mn\times Fe\times {F}_{v}\right)}^{1/D}}\right) \\ & =\frac{1}{D}\left[ln\left(\frac{N}{N}\right)+ln\left(\frac{N}{P}\right)+ln\left(\frac{N}{K}\right)+ln\left(\frac{N}{Ca}\right)+ln\left(\frac{N}{Mg}\right)+ln\left(\frac{N}{S}\right)+ln\left(\frac{N}{B}\right)+ln\left(\frac{N}{Cu}\right)+ln\left(\frac{N}{Zn}\right)+ln\left(\frac{N}{Mn}\right)+ln\left(\frac{N}{Fe}\right)+ln\left(\frac{N}{{F}_{v}}\right)\right]\end{aligned}$$

Because the $clr$ values are computed about the geometric mean, the sum of $clr$ values is zero. The mean $clr$ value for component *i* can be back transformed into its concentration value ${x}_{i}$ as follows:

1. $${exp}_{{x}_{i}}=exp\left({clr}_{{x}_{i}}\right)$$
2. $${x}_{i}=\frac{{\kappa \times exp}_{{x}_{i}}}{{\sum }_{i=1}^{D}{exp}_{{x}_{i}}}$$

Where *exp* is the exponential transformation of the centered log ratio and $\kappa$ is the unit of measurement (e.g., 1000 g kg^−1^) to force closure to the measurement unit (here, g kg^−1^).

The *clr* variables have Euclidean geometry. The diagnosed composition can thus be compared to the composition of the closest successful neighbors (high-yielding and nutritionally balanced specimens) as the ones showing the shortest Euclidean distance $\varepsilon$ from the diagnosed composition computed as follows:

$$\varepsilon =\sqrt{\sum \limits_{k=1}^{D}{\left({clr}_{i}-{clr}_{i}^{*}\right)}^{2}}$$

where ${clr}_{i}$ is the $clr$ value of component $i$ of the diagnosed composition, and ${clr}_{i}^{*}$ is the *clr* value of component $i$ of a close successful compositional neighbor. In Brazil, *clr* indices are widely used to diagnose the plant nutrient status^72^ using $clr$ reference values^73^. Tissue nutrient indices (${I}_{{x}_{i}}$) are differences between diagnosed $clr$ value (${clr}_{{x}_{i}}$) and the $clr$ mean (${clr}_{{x}_{i}}^{*}$) for true negative specimens (TN) weighted by the standard deviation (${SD}_{{x}_{i}}^{*}$), computed as follows^74^:

$${I}_{{x}_{i}}=\frac{{clr}_{{x}_{i}}-{clr}_{{x}_{i}}^{*}}{{SD}_{{x}_{i}}^{*}}$$

Nutrient indices can be displayed in a histogram to indicate relative excess or shortage of nutrients, respectively. The nutrient standards for high-yielding and nutritionally balanced specimens can be computed regionally (e.g., across the surveyed area), or from a selection of close compositional neighbors.

#### Machine learning models

Several machine learning (ML) models can be tested using the Orange data mining freeware vs. 3.29. In the ML models, the target variable was marketable bulb yield. Features were climatic indices, nutrient dosage, soil and tissue analyses, cultivar, crop establishment (direct seeding or manual transplanting), soil management, municipality, climatic indices, date of stand establishment, and harvest date (source), as described in Table 6.

**Table 6:** List of candidate features in the onion data set of Santa Catarina state, Brazil.

Feature group	Candidates
1. Climate	Cumulated precipitations, standardized Shannon Diversity Index(SDI), temperature (minimum, maximum), cumulated degree-days (> 5°), planting or seeding Julian day reflecting photoperiod, length of the growing season, seasonal observations (below normal, normal, above normal conditions, excess, hail, frost)
2. Management	Stand establishment (transplanting, direct seeding), tillage practice (conventional, no-till), cultivar (‘Caeté’, ‘‘Empasc 352 Bola Precoce’, ’Epagri 362 Crioula Alto Vale’, ‘Mulata’, ‘Omega’, ‘SCS373 Valessul’), population density, preceding crop
3. Soil	Soil classification, soil test (clay, organic matter, pH, P, K, Ca, Mg, S, B, Cu, Zn, Mn, Fe), cation exchange capacity (CEC), base saturation of CEC
4. Tissue	N, S, P, K, Ca, Mg, S, B, Cu, Zn, Mn, Fe
5. Fertilization	Fertilizer source, dosage and timing and placement

Summaries of tissue and soil test results used as features are presented in Table 3 and Supplementary Material S3, respectively. Other features were managerial or climatic. ‘Empasc 352 Bola Precoce’ and ‘SCS373 Valessul’ are short-day cultivars requiring 11–13 h to initiate bulbification. Median-day cultivars requiring 13–15 h to initiate bulbation were ‘’Epagri 362 Crioula Alto Vale, ‘Mulata’, ‘Omega’ and ‘Caeté’. We discarded ‘Bola Precoce’ specimens because tissue analysis for sulfur was absent. Onions were seeded or transplanted. Crops were established by direct seeding or were transplanted manually. Stand establishment, soil management and previous crops are reported in Supplementary Material S4. Previous crops were black oat (*Avena sativa*), millet (*Pennisetum glaucum*), sweet potato (*Ipomoea batatas*), tobacco (*Nicotiana tabacum*), corn (*Zea mays*), cowpea (*Vigna unguiculata* (L.) Walp.), velvet bean (*Mucuna aterrima*) and millet (*P. glaucum*). Preceding crops varied among years and locations. Climatic conditions varied widely at experimental sites as shown in Supplementary Material S5. The importance of features in relation to bulb yield was measured as RReliefF ranking scores^75^. The RReliefF algorithm computes a difference between actual and predicted values in regression problems based on the nearest neighbor paradigm after considering feature interactions.

Two decision-tree ML regression models were tested among more than 100 variants commonly used in soil science^40,66^, i.e., random forest and extreme gradient boosting, both available in the Orange Data Mining freeware v. 3.39.0 programmed in the Python language (University of Ljubljana, Ljubljana, Slovenia). The Python algorithms are encoded into icons and arrows. The scheme of icons and arrows is presented in Supplementary Materials S1 and S2. There were several missing data in the dataset (13%). The dataset was thus rebalanced by model-based imputation using the random forest imputation method^76,77^.

Decision-tree models separate two subsets recursively about cutoff points that minimize the variance of the target variable until a minimum number of instances is reached. Random forest and extreme gradient boosting are structurally different. Random forest is a bagging model that averages predictions made by sampling with replacement. We selected 10 trees per bag at each run. Extreme gradient boosting is a variant of the tree-based ensemble gradient boosting method that combines weak predictive models to minimize prediction error. The extreme gradient boosting creates and adds trees of learners sequentially to correct the weakness of the preceding estimators. We selected 100 trees as basic property.

The partition between the training and testing datasets was conducted by stratified random sampling. The population of data comprised subgroups of categorial variables or strata. Data were randomly sampled within each strata. This avoids sampling data from the same strata during the partition between the training set and testing sets. Otherwise, complete random sampling leads to model overfitting. The train/test partitions were repeated 100 times, and model accuracy was averaged. The accuracy of the partition between the training and the testing sets reached a plateau at 70:30. Such partition was thus selected to process the data.

The regression ML model returns a relationship between the actual and the predicted starget variable. Model accuracy is reported as root mean squared error (RMSE), median absolute error (MAE), and coefficient of determination or R^2^. Model strength is substantial if R^2^ is > 75%^78^. The classification mode returns a confusion matrix where specimens are classified into four quadrants: true negative (yield above cutoff, nutritionally balanced composition), false negative (yield below cutoff, nutritionally balanced composition), false positive (yield above cutoff, nutritionally imbalanced composition) and true positive (yield below cutoff, nutritionally imbalanced composition). True negative specimens provided a set of successful features to compute tissue nutrient standards amongst others. The accuracy of the classification model is measured by the area under curve and the classification accuracy.

### Supplementary Information

Supplementary Information.

## Data Availability

All data and the model used for analysis are available at Zenodo DOI 10.5281/zenodo.10615658. The experimental research and field studies on plants in this work strictly comply with relevant institutional, national and international guidelines and legislation.
