# Supplementary material for: Feature-specific nutrient management of onion (Allium cepa) using machine learning and compositional methods
Source: Sci Rep. 2024 Mar 12;14:6034. doi: 10.1038/s41598-024-55647-9 (PMC10933362; doi:10.1038/s41598-024-55647-9)
Supplement: Supplementary file 1 — Supplementary Information. [file 41598_2024_55647_MOESM1_ESM.docx]

**Supplementary material S1.** Scheme of the Orange Data Mining software to run data imputation, input files, select variables and solve relationships in regression (scatter plot) or classification (confusion matrix) modes.


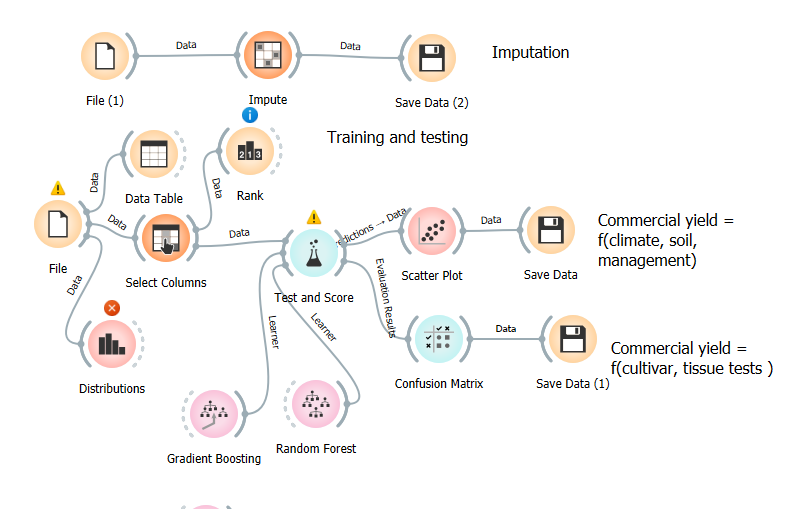


**Supplementary material S2**. Scheme of the Orange Data Mining software to make predictions after connecting the Random Forest and Gradient Boosting icons to the data selection icon in Supplementary material S2. The file comprises data collected at unseen sites (not present in the training and testing sets) where a gradient of fertilizer rates is tested given that features are those provide by the in growers. Tests in growers’ fields are called ‘universality tests’ to verify model’s ability to generalize.


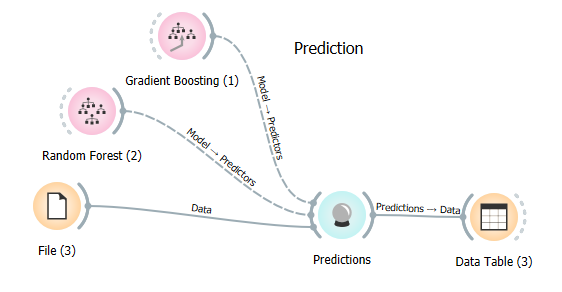


**Supplementary material S3.** Results of soil analysis.

| **Variable** | **Unit** | **Minimum** | **Mean** | **Maximum** |
| --- | --- | --- | --- | --- |
| Clay content | % | 15 | 38 | 67 |
| Soil organic matter | % | 2.2 | 3.5 | 5.8 |
| pH_water_ | - | 5.1 | 5.9 | 6.9 |
| Soil test P | mg dm^-3^ | 3.2 | 22.0 | 77.7 |
| Soil test K | mg dm^-3^ | 59 | 192 | 936 |
| Soil test Ca | cmol_c_ dm^-3^ | 5.0 | 7.4 | 15.3 |
| Soil test Mg | cmol_c_ dm^-3^ | 2.2 | 3.3 | 5.9 |
| Cation exchange capacity | cmol_c_ dm^-3^ | 11.7 | 16.3 | 25.0 |
| Base saturation | % | 55.8 | 76.2 | 88.8 |
| Soil test S | mg dm^-3^ | 12.5 | 22.5 | 96.6 |
| Soil test Zn | mg dm^-3^ | 2.0 | 2.8 | 10.3 |
| Soil test Cu | mg dm^-3^ | 0.4 | 5.0 | 13.1 |
| Soil test Mn | mg dm^-3^ | 0.1 | 18.3 | 64.2 |
| Soil test B | mg dm^-3^ | 0.0 | 1.0 | 1.4 |
| Soil test Fe | mg dm^-3^ | 28 | 105 | 246 |

**Supplementary material S4.** Cultivar and preceding crop

| **Year** | **#site** | **Cultivar** | **Bulb color** | **Crop establisihment** | **Soil management** | **Preceding crop** | **Nutrients rates (kg/ha)** | | |
| --- | --- | --- | --- | --- | --- | --- | --- | --- | --- |
|  |  |  |  |  |  |  | **N** | **P** | **K** |
| 2007 | 1 | ‘Bola Precoce’ | Yellow | Machine transplanting | No-till | Black oat (*Avena sativa*) | 0, 50, 100, 200 | 52 | 75 |
| 2008 | 2 | ‘Bola Precoce’ | Yellow | Machine transplanting | No-till, conventional | Sweet potato (*Ipomoea batatas*) | 0, 50, 100, 200 | 52 | 75 |
|  | 3 | ‘Bola Precoce’ | Yellow | Machine transplanting | No-till, conventional |  | 0, 50, 100, 200 | 70 | 75 |
| 2009 | 4 | ‘Bola Precoce’ | Yellow | Machine transplanting | Conventional | Millet (*Pennisetum glaucum*) | 0, 50, 100, 200 | 70 | 75 |
|  | 5 | ‘Bola Precoce’ | Yellow | Machine transplanting | Conventional | Tobacco (*Nicotiana tabacum*) | 0, 32, 64,75, 83, 128 | 0, 44, 87, 175, 349 | 0, 42, 75, 83, 167, 333, 667 |
| 2011 | 6 | ‘Bola Precoce’ | Yellow | Machine transplanting | No-till, conventional | Millet (*Pennisetum glaucum*) | 0, 50, 100, 200 | 70 | 75 |
|  | 7 | ‘Bola Precoce’ | Yellow | Machine transplanting | No-till, conventional | Millet (*Pennisetum glaucum*) | 100 | 0, 44, 87, 175, 349 | 0, 83, 167, 333, 667 |
| 2012 | 8 | ‘Bola Precoce’ | Yellow | Direct seeding | Conventional | Millet (*Pennisetum glaucum*) | 0, 60, 120, 180, 240 | 180 | 83 |
|  | 9 | ‘Crioula Alto Vale’ | Yellow | Direct seeding | Conventional | Millet (*Pennisetum glaucum*) | 0, 60, 120, 180, 240 | 79 | 83 |
| 2013 | 10 | ‘Bola Precoce’ | Yellow | Direct seeding | Conventional | Millet (*Pennisetum glaucum*) | 0, 60, 120, 180, 240 | 79 | 75 |
|  | 11 | Crioula Alto Vale’ | Yellow | Direct seeding | Conventional | Millet (*Pennisetum glaucum*) | 0, 60, 120, 180, 240 | 79 | 75 |
| 2014 | 12 | ‘Bola Precoce’ | Yellow | Direct seeding | Conventional | Millet (*Pennisetum glaucum*) | 0, 60, 120, 180, 240 | 79 | 75 |
|  | 13 | ‘Crioula Alto Vale’ | Yellow | Direct seeding | Conventional | Millet (*Pennisetum glaucum*) | 0, 60, 120, 180, 240 | 79 | 75 |
| 2015 | 14 | ‘Bola Precoce’ | Yellow | Manual transplanting | No-till | Mixture of *Pennisetum glaucum* (30 kg ha^-1^) and *Vigna unguiculata* (L.) Walp. (40 kg ha^-1^) | 0,75, 125 | 0, 26, 35, 55 | 0, 75, 117, 195, 277 |
| 2016 | 15 | ‘Bola Precoce’ | Yellow | Direct seeding | Conventional | Maize (*Zea Mays*) | 0, 150 | 0, 122 | 0, 50 |
|  | 16 | ‘Mulata’ | Yellow | Direct seeding | No-till | Black oat (*Avena sativa*) | 20, 90, 160, 230, 300 | 161 | 50 |
| 2017 | 17 | ‘Bola Precoce’ | Yellow |  |  |  | 150 | 122 | 75 |
|  | 18 | ‘Omega’ | Yellow | Direct seeding | No-till, conventional | Black oat (*Avena sativa*) | 20, 90, 160, 230, 300, 370 | 161 | 50 |
|  | 19 | ‘Omega’ | Yellow | Direct seeding | No-till, conventional | Black oat (*Avena sativa*) | 20, 90, 160, 230, 300, 370 | 131 | 25 |
| 2018 | 20 | ‘Bola Precoce’ | Yellow | Manual transplanting | No-till | Mixture of *Mucuna aterrima* (40 kg ha^-1^ ha) and *Pennisetum glaucum* (30 kg ha^-1^) | 280 | 65 | 75 |
|  | 21 | ‘Omega’ | Yellow | Direct seeding | No-till | Black oat (*Avena sativa*) | 381 | 17 | 183 |
| 2019 | 22 | ‘Valessul’ | Yellow | Machine transplanting | Conventional | Millet (*Pennisetum glaucum*) | 140 | 61 | 125 |
|  | 23 | ‘Omega’ | Yellow | Direct seeding | No-till | Black oat (*Avena sativa*) | 330 | 196 | 183 |
| 2020 | 24 | ‘Valessul’ | Yellow | Machine transplanting | No-till, conventional | Millet (*Pennisetum glaucum*) | 0, 180 | 96 | 125 |
|  | 25 | ‘Valessul’ | Yellow | Direct seeding | No-till | Black oat (*Avena* *sativa*) | 180 | 61 | 125 |
|  | 26 | ‘Caeté’ | Purple | Direct seeding | No-till | Black oat (*Avena* *sativa*) | 351 | 124 | 196 |

**Supplementary Material S5.** Summary of climatic conditions during the growing season at experimental sites

| **Year** | **Duration** | **Rainfall** | **Shannon diversity index** | **Degree-days > 5°C** | **Minimum temperature** | | **Maximum temperature** | | **Crop** |
| --- | --- | --- | --- | --- | --- | --- | --- | --- | --- |
|  |  |  |  |  | **minima** | **maxima** | **minima** | **maxima** | **scouting** |
|  | d | mm | unitless | °C | | | | |  |
|  | Ituporanga† and Atalanta‡ experimental sites | | | | | | | |  |
| 2007† | 115 | 604 | 0.66 | 1344 | -0.8 | 21.0 | 10.6 | 31.6 | Normal |
| 2008† | 123 | 835 | 0.77 | 1427 | 0.0 | 19.2 | 13.0 | 32.4 | Rainfall Normal normal or  above average |
| 2009† | 115 | 749 | 0.70 | 1395 | 4.0 | 21.0 | 12.6 | 37.4 | Rainfall above average |
| 2009† | 121 | 796 | 0.70 | 1446 | -0.6 | 21.0 | 10.8 | 37.4 | Rainfall above average |
| 2011† | 120 | 853 | 0.68 | 1382 | -1.8 | 17.4 | 11.2 | 34.6 | Rainfall below average |
| 2011† | 126 | 908 | 0.68 | 1412 | -1.8 | 17.4 | 11.2 | 34.6 | Rainfall below average |
| 2012† | 176 | 670 | 0.69 | 1524 | 0.3 | 21.0 | 11.8 | 31.9 | Rainfall below average |
| 2012† | 181 | 703 | 0.70 | 1684 | 0.3 | 21.0 | 11.8 | 32.8 | Rainfall below average |
| 2013† | 180 | 989 | 0.71 | 1882 | -2.9 | 19.6 | 7.6 | 32.0 | Rainfall below average |
| 2013† | 185 | 992 | 0.71 | 1967 | -2.9 | 19.6 | 7.6 | 33.4 | Rainfall below average |
| 2014† | 181 | 1026 | 0.69 | 2112 | 1.0 | 21.2 | 13.5 | 33.5 | Rainfall above average |
| 2014† | 189 | 1065 | 0.70 | 2248 | 1.0 | 21.2 | 13.5 | 33.5 | Rainfall above average |
| 2015† | 128 | 822 | 0.73 | 1594 | 3.2 | 20.0 | 14.0 | 33.2 | Excessive rainfall |
| 2016‡ | 164 | 646 | 0.68 | 1687 | -2.0 | 19.0 | 13.2 | 30.9 | Normal |
| 2017† | 108 | 331 | 0.63 | 1311 | -0.8 | 19.6 | 13.7 | 33.5 | Normal |
| 2018† | 125 | 470 | 0.68 | 1442 | 0.8 | 18.8 | 13.3 | 30.7 | Normal |
| 2019† | 109 | 361 | 0.71 | 1365 | -0.4 | 20.4 | 13.3 | 36.3 | Rainfall below average, hail |
| 2020† | 123 | 378 | 0.74 | 1501 | 1.6 | 20.5 | 11.9 | 31.5 | Rainfall below average |
| 2020† | 125 | 399 | 0.75 | 1516 | 1.6 | 20.5 | 11.9 | 31.5 | Rainfall below average |
|  | Lebon Regis and Caçadorϯ experimental sites | | | | | | | |  |
| 2016¶ | 158 | 715 | 0.72 | 1624 | -4.2 | 19.0 | 8.6 | 31.2 | Normal |
| 2017¶ | 157 | 563 | 0.69 | 1857 | 0.1 | 19.1 | 13.7 | 31.7 | Normal |
| 2017ϯ | 163 | 543 | 0.67 | 1826 | -2.9 | 18.8 | 8.3 | 31.7 | Normal |
| 2018¶ | 156 | 570 | 0.71 | 1738 | -0.5 | 19.0 | 9.1 | 31.6 | Normal |
| 2019¶ | 156 | 444 | 0.66 | 1880 | -2.3 | 20.9 | 9.0 | 34.0 | Normal |
| 2020¶ | 161 | 510 | 0.68 | 1842 | -4.6 | 19.0 | 8.8 | 35.2 | Normal |
